# New Mode of Treating Epiphora

**Published:** 1851-11

**Authors:** 


*New mode of treating Epiphora.* By Mr. Bowman.—This plan consists in slitting up the canal from the punctum on the conjunctival aspect, so as to carry backwards the orifice at which the tears are received on to the mucous membrane near the caruncle; and Mr. Bowman finds that the tears are in fact taken up by the remaining portion of the canal, while the end towards the punctum is converted into a groove. For the cases of obstruction from injury or other cause he suggests a modification of this operation, by which the canal between the obstruction and the sac may be slit up for some way, so as to receive the tears at a new opening. The cases to which these new operations are applicable have been for the most part abandoned by surgeons as incurable.—*Medical Times.*

